# Clinical Features of Acute Chikungunya Virus Infection in Children and Adults during an Outbreak in the Maldives

**DOI:** 10.4269/ajtmh.21-0189

**Published:** 2021-08-02

**Authors:** Hisham Ahmed Imad, Juthamas Phadungsombat, Emi E. Nakayama, Keita Suzuki, Ahmed Mifthah Ibrahim, Aminath Afaa, Aminath Azeema, Aminath Nazfa, Aminath Yazfa, Anoosha Ahmed, Athifa Saeed, Azna Waheed, Fathimath Shareef, Mohamed Moinul Islam, Shausha Mohamed Anees, Sana Saleem, Aminath Aroosha, Ibrahim Afzal, Pornsawan Leaungwutiwong, Watcharapong Piyaphanee, Weerapong Phumratanaprapin, Tatsuo Shioda

**Affiliations:** ^1^ Mahidol-Osaka Center for Infectious Diseases, Faculty of Tropical Medicine, Mahidol University, Bangkok, Thailand;; ^2^ Department of Viral Infections, Research Institute for Microbial Diseases, Osaka University, Osaka, Japan;; ^3^ POCT Products Business Unit, TANAKA Kikinzoku Kogyo, Hiratsuka, Japan;; ^4^ Indira Gandhi Memorial Hospital, Malé, Maldives;; ^5^ Health Protection Agency, Ministry of Health, Malé, Maldives;; ^6^ Tropical Medicine Diagnostic Reference Laboratory, Faculty of Tropical Medicine, Mahidol University, Bangkok, Thailand;; ^7^ Department of Microbiology and Immunology, Faculty of Tropical Medicine, Mahidol University, Bangkok, Thailand;; ^8^ Department of Clinical Tropical Medicine, Faculty of Tropical Medicine, Mahidol University, Bangkok, Thailand

## Abstract

The chikungunya virus is an arthritogenic arbovirus that has re-emerged in many tropical and subtropical regions, causing explosive outbreaks. This re-emergence is due to a genomic polymorphism that has increased the vector susceptibility of the virus. The majority of those infected with chikungunya virus exhibit symptoms of fever, rash, and debilitating polyarthralgia or arthritis. Symptoms can persist for weeks, and patients can relapse months later. Fatalities are rare, but individuals of extreme age can develop severe infection. Here, we describe the 2019 outbreak, the second-largest since the virus re-emerged in the Maldives after the 2004 Indian Ocean epidemic, in which a total of 1,470 cases were reported to the Health Ministry. Sixty-seven patients presenting at the main referral tertiary care hospital in the Maldives capital with acute undifferentiated illness were recruited following a negative dengue serology. A novel point-of-care antigen kit was used to screen suspected cases, 50 of which were subsequently confirmed using real-time reverse transcription–polymerase chain reaction. We describe the genotype and polymorphism of Maldives chikungunya virus using phylogenetic analysis. All isolates were consistent with the East Central South African genotype of the Indian Ocean lineage, with a specific E1-K211E mutation. In addition, we explored the clinical and laboratory manifestations of acute chikungunya in children and adults, of which severe infection was found in some children, whereas arthritis primarily occurred in adults. Arthritides in adults occurred irrespective of underlying comorbidities and were associated with the degree of viremia.

## INTRODUCTION

Chikungunya virus (CHIKV) is a mosquito-borne re-emerging *Alphavirus* of the *Togaviridae* family within the Semliki forest antigen complex. The virus was first discovered in Africa and later spread to Asia, giving rise to three distinct lineages^1^^–^^4^ Decades later an outbreak occurred in eastern Africa that is believed to have led to a spillover of the virus to the Indian Ocean islands, resulting in the emergence of a CHIKV sublineage, and to date a number of distinct, lineage-specific novel strains have been isolated and characterized.^5^^,^^6^ A CHIKV genomic mutation that increased the susceptibility of *Aedes albopictus* to CHIKV has been associated with the rapid dissemination of CHIKV in the Indian Ocean region.^7^

With an incubation period of 2–12 days, the majority of cases are symptomatic.^8^ The most typical and characteristic clinical manifestations of CHIKV infection include high-grade fever associated with arthralgia and rash.^9^ Arthralgia and arthritis are reportedly the most debilitating symptoms, which can occur in 87% of adults and in 30–50% of children.^10^^–^^13^ Although individuals of all ages are susceptible to the virus, the arthritogenic manifestations of arthralgia are less prominent in children, whereas females appear to be at higher risk of developing incapacitating symptoms.^14^ Severe forms of infection-associated neuro-invasive disease can occur in children, and other atypical manifestations or fatality can occur in individuals with underlying comorbidities.^15^^–^^23^

The Maldives, a tropical island archipelago in the Indian Ocean, experienced a CHIKV outbreak in 2005–2006 involving anthropophilic vector *Aedes* species mosquitos that inhabit these small coral islands.^24^ The last major outbreak of CHIKV was reported in 2006 and involved 11,879 cases. Infection was confirmed in 67 of these cases and resulted in six fatalities.^25^

We describe the initial months of the resurgence of an outbreak of CHIKV infection that began in late 2018 in the Maldives. In the present study, we describe the clinical manifestations and characteristics of this outbreak of acute CHIKV infection in a patient cohort that included children and adults. We also describe the genotype and polymorphisms of Maldives CHIKV based on phylogenetic analyses. To the best of our knowledge, this was the second-largest outbreak ever documented in the Maldives, with the longest epidemic period since the explosive outbreak in the Indian Ocean over a decade ago.

## MATERIALS AND METHODS

We conducted a prospective study during a CHIKV outbreak in the Maldives that began in late 2018 at Indira Gandhi Memorial Hospital, located in the capital city of Malé, which serves as the central referral center in the country. Informed consent, and assent where necessary, was obtained from each study participant. The inclusion criteria included all patients who presented to the hospital within 5 days after having developed symptoms characteristic of acute CHIKV infection and were febrile with a body temperature not lower than 38.5°C. In addition, any study participants with a positive antigen or serology test for dengue were excluded. A rapid immunochromatographic test kit was used to detect the CHIKV envelope protein 1 (E1) antigen as a point of screening during recruitment, and all cases were subsequently subjected to real-time reverse transcription–polymerase chain reaction (RT-PCR) analysis for confirmation of CHIKV infection. We defined confirmed cases as those with positive results by real-time RT-PCR analysis for CHIKV. In view of the possibility of co-infections, real-time RT-PCR was performed on all 50 specimens, which excluded possible co-infection with the dengue or Zika viruses. Data regarding clinical and laboratory features were collected anonymously at the time of presentation using a standardized case record sheet, from which an electronic data set was created for analysis. Leftover serum samples from routine investigation performed at the hospital were collected from the hospital central laboratory. All clinical specimens were transported from the Maldives to Thailand for molecular testing for CHIKV and phylogenetic analysis at Mahidol-Osaka Center for Infectious Diseases. Epidemiologic data were obtained via formal request from the Health Protection Agency at the Ministry of Health in the Maldives.

### Immunochromatography analysis of CHIKV antigen.

The prototype kit was developed at Mahidol-Osaka Center for Infectious Diseases; details regarding the immunochromatography kit have been described previously, including data pertaining to validation of kit performance.^26^^–^^28^ Briefly, leftover serum samples from routine investigation performed at the hospital were collected from the hospital central laboratory. A 30-μL aliquot of each serum sample was analyzed for the antigen by mixing the serum with 60 μL of extraction buffer in a microtube. The immunochromatography strip was then placed in the mixed solution of serum and buffer. Results were interpreted by the appearance of control and test bands assessed after 15 min.

### RT-PCR analysis.

The CHIKV genome was quantified using previously described methods.^29^ Briefly, RNA was extracted using a viral RNA extraction kit (QiAmp Viral RNA mini kit; Qiagen, Hilden, Germany). SYBR green (Bio-Rad, Hercules, CA) quantitative RT-PCR was used to detect CHIKV by targeting a 120-bp region of the genome encoding the E1 protein. The primers used in the study were 5'-CTCATACCGCATCCGCATCAG-3' (forward) and 5'-ACATTGGCCCCACAATGAATTTG-3' (reverse). A standard curve was constructed using RNA prepared from a CHIKV isolate obtained in an earlier study and included six dilutions containing 10^1^–10^6^ plaque-forming units (PFU)/mL; the detection limit was approximately 10^2^ PFU/mL.

### CHIKV E1 sequencing and phylogenetic analysis.

The nucleotide sequence encoding Maldives CHIKV E1 protein was determined by Sanger sequencing using the extracted RNA from CHIKV-positive real-time RT-PCR samples. Briefly, 4 µL of RNA was mixed with a specific reverse primer that binds to the end of the CHIKV genome (3RT),^5^^,^^30^ along with deoxynucleoside triphosphate, buffer, MgCl_2_, and dithiothreitol according to the manufacturer’s protocol. The RNA was converted into cDNA using a Superscript III first-strand synthesis system (Invitrogen, Carlsbad, CA). Next, 2 µL of cDNA was further amplified using Primestar GXL DNA polymerase (Takara, Japan) with a forward primer (chf23) and reverse primer (3RT) to obtain a 2.0-kb sequence covering 1,317 bp of the CHIKV E1–encoding region. The amplicons were purified (Nucleospin, Macherey-Nagel, Germany) and sequenced (Macrogen, Seoul, Korea) using the primers chf24 and chr25.^31^ The resulting sequences were aligned to the sequence of the reference CHIKV African prototype strain S27 (NC_004162.2) using AliView V1.26,^32^ and the consensus sequence of the CHIKV E1 region (1,317 bp) was manually extracted and deposited in GenBank (accession numbers LC603810–LC603843). The sequence similarity of the newly obtained sequences was examined using NCBI Nucleotide Blast. The E1 nucleotide sequence dataset for ECSA CHIKV was prepared and a phylogenetic tree constructed according to the maximum-likelihood (ML) method using IQTREE^33^ under TIM2e+G4 and 1,000 replicated ultrafast bootstrap analysis. The ML tree was visualized using FigTree v1.4.4 (http://tree.bio.ed.ac.uk/software/figtree/).

### Data analysis.

All available data from the 50 laboratory-confirmed cases were analyzed. We also analyzed clinical and laboratory data for adults and children. Adult patients with underlying comorbidities were segregated into group A, and those without known comorbidities were segregated into group B. The clinical and laboratory findings of children with severe CHIKV infection versus children with nonsevere CHIKV infection were also analyzed. Severe infection was classified as presentation with encephalitis. Lastly, we compared the clinical and laboratory findings of patients with confirmed CHIKV infection with those of our previous dengue patient cohort. We classified all patients who presented on days 1 and 2 of illness as group A, patients who presented on day 3 of illness as group B, and patients who presented on days 4 and 5 of illness as group C. All analyses were performed using Statistical Package for the Social Sciences software. Distributive frequencies of clinical manifestations were estimated by all-group analysis. All categorical variables were tested for observed frequencies using the χ^2^ test. Wilcoxon and Mann-Whitney tests were used for nonparametric testing. Nonparametric Spearman correlation coefficients were calculated to analyze correlations. Tables were created using Microsoft Excel, and graphs were prepared using Graph Pad Prism software. The kit’s sensitivity was calculated by dividing the true positive with true positive and false negative multiplied by 100. Specificity was calculated by dividing the true negative with true negative and false positive multiplied by 100. Positive predictive value was calculated by dividing the true positive by true positive and false positive multiplied by 100. Negative predictive value was calculated by dividing the true negative by true negative and false negative multiplied by 100.

## RESULTS

An outbreak of CHIKV infection occurred in the Maldives in late 2018. In the Maldives, CHIKV infection is classified as a “notifiable’ infectious disease. The Health Protection Agency serves as a sentinel for surveillance and monitoring of diseases in the Maldives. Based on their data of reported CHIKV infection cases from the beginning of January 2019 to the first week of December 2019, there were 1,470 reported cases of CHIKV infection (Supplemental Figure 1). The incidence rate of acute CHIKV infection across the country is shown in Supplemental Figure 2.

The average incidence rate was 3.2 per 1000 individuals, with comparatively higher incidence rates of 18.6 and 16.2 per 1000 individuals in the North Gaaf and Raa atolls. Forty-two percent of cases involved females, and 58% involved males; a total of 24.4% of cases involved children, and 75.6% involved adults. The distribution of all reported cases by age is shown in Supplementary Figure S3. The median age for all reported cases was 33 years, with an interquartile range (IQR) of 19–46 years. The median (IQR) age of adult females and males was 38 (29–50) and 38 (30–50) years, respectively. The median (IQR) age of female and male children was 9 (5–12) and 9 (6–12) years, respectively. The majority of patients (93.5%) were Maldivians, and 6.5% were foreigners.

### Demographic and clinical findings of the study group.

Sixty-seven patients were prospectively recruited from April until June 2019. Among these patients, 50 were positive for CHIKV by real-time RT-PCR analysis. The rapid point-of-care diagnostic kit demonstrated a sensitivity of 70% and a specificity of 81.25% in these 67 cases, as shown in Supplemental Table 1. Among the 50 laboratory-confirmed cases of acute CHIKV infection, 36% were female and 64% male. There were 10 children under the age of 18 years and 40 adults. The median age was 9 years (IQR: 5–13 years) in the children and 50 years (IQR: 36–68 years) in the adults. More patients were in the 31–40 and 61–70 year age groups. More females in their 30s were affected than males. Underlying medical conditions such as diabetes, hypertension, and ischemic heart disease were only present in adults.

**Figure 1.**
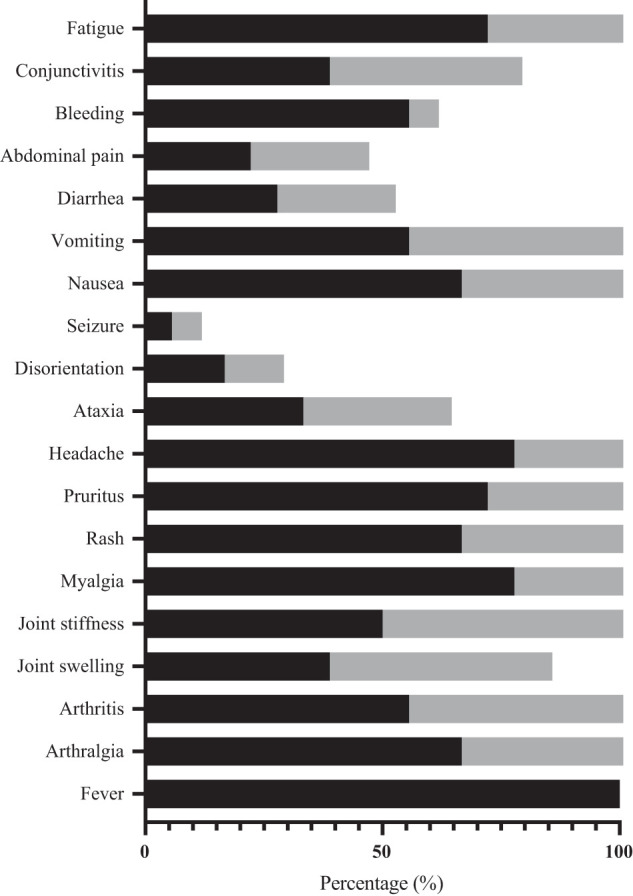
Clinical manifestations of acute CHIKV infection (*N* = 50). Black represents females; grey represents males.

**Figure 2.**
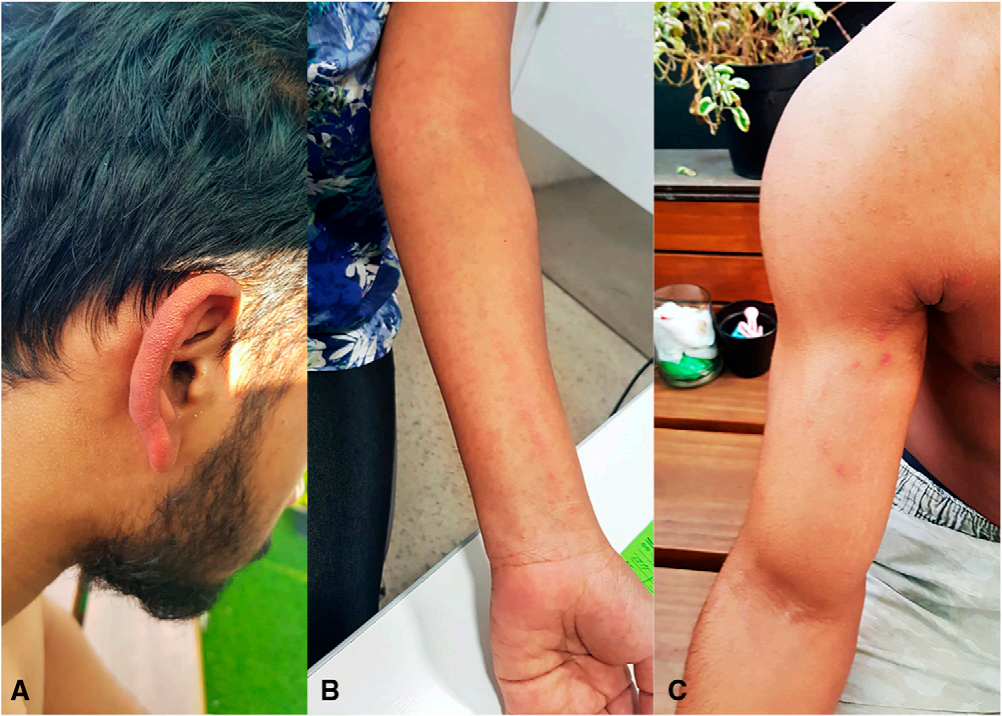
Observed cutaneous manifestations. (**A**) Milians ear sign. (**B**) Maculopapular rash. (**C**) Scar phenomenon. This figure appears in color at www.ajtmh.org.

Fever was present at the time of presentation in all cases. Arthralgia and myalgia were the most common manifestations, reported in up to 82% of cases, followed by headache (74%), fatigue (66%), nausea (62%), vomiting (60%), arthritis (58%), and rash (54%), as shown in Figure 1. Figure 2 shows the cutaneous involvement observed in cases in this study. Arthralgia was more common in males (*P* = 0.034; adjusted *P* = 0.612), whereas bleeding (*P* < 0.000; adjusted *P* < 0.000) and pruritus (*P* = 0.032; adjusted *P* = 0.576) were more common in females, although the differences in arthralgia and pruritus did not reach statistical significance after adjustment for multiple comparisons. Overall, the majority of cases exhibited typical clinical characteristics consistent with acute CHIKV infection. Only three pediatric cases involved encephalitis, and one adult developed sepsis leading to disseminated intravascular coagulation. Nine patients were hospitalized after presentation, including the three children with encephalitis and adults with excruciating headache and disorientation. Comparison of clinical and laboratory profile at the time of presentation of these chikungunya cases with those of dengue cases in our previous study is shown in Supplemental Table 4.^34^

**Figure 3.**
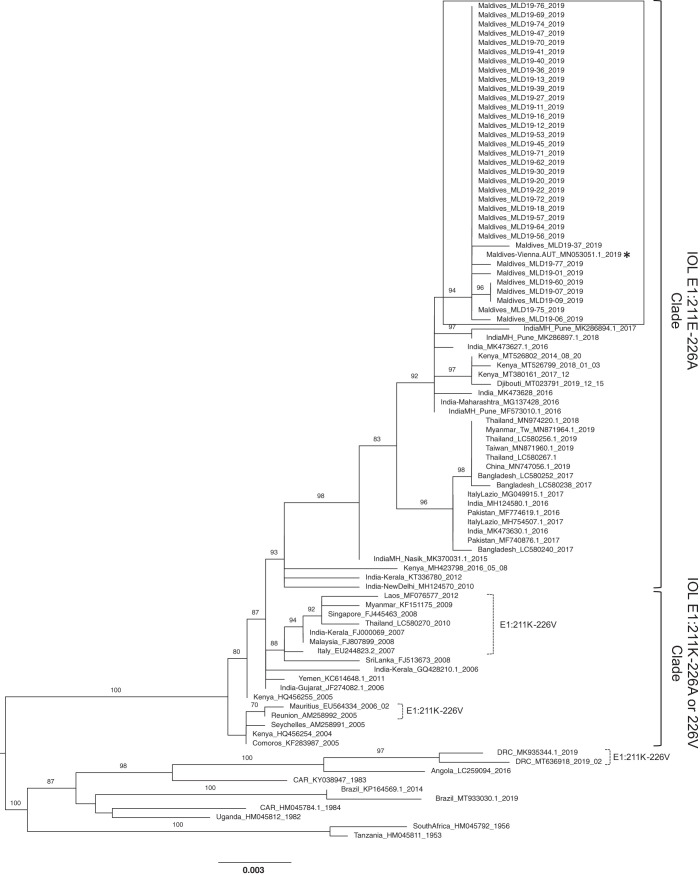
Phylogenetic analysis of the Maldives ECSA CHIKVs circulating in 2019.

### Analysis of differences in clinical findings between children and adults.

Because the literature on chikungunya in children is limited with a very few studies comparing the clinical findings in children and adults, we compared clinical manifestations in children with those of adults in our patient cohort. The differences in clinical characteristics of acute chikungunya in children and adults are shown in Table 1. Arthralgia was present in both children (70%) and adults (80%). Arthritis was significantly more frequent in adults (70%) than children (10%) (*P* = 0.001; adjusted *P* = 0.025). Similarly, swelling of the joints and stiffness tended to be more frequent in adults (*P* = 0.015; adjusted *P* = 0.375 and *P* = 0.002; adjusted *P* = 0.375, respectively), although these differences did not reach statistical significance after adjustment for multiple comparisons. We also noted that ataxia only presented in adults (40%), whereas 30% of children developed seizures. No other significant differences in clinical manifestations were observed between children and adults.

**Table 1:** Clinical characteristics of acute chikungunya in children and adults (*N* = 50)

Clinical characteristics	Children (*N* = 10)	Adults (*N* = 40)	*P* value	Adjusted *P* value
Duration of illness, days	2 (1–3)*	3 (2–3)	0.159	1.000
Females	2 (20)	16 (40)	0.239	1.000
Males	8 (80)	24 (60)		
Age, years	9 (5–13)	50 (36–69)	**0.000**	**0.000**
Comorbidities	0	15 (37.5)	**0.021**	0.525
Hospitalization	3 (30)	6 (15)	0.269	1.000
Fever	10 (100)	40 (100)	NA	NA
Arthralgia	7 (70)	34 (85)	0.269	1.000
Arthritis	1 (10)	28 (70)	**0.001**	**0.025**
Joint swelling	1 (10)	21 (52.5)	**0.015**	0.375
Joint stiffness	1 (10)	26 (65)	**0.002**	**0.050**
Myalgia	7 (70)	33 (82.5)	0.377	1.000
Headache	6 (60)	31 (77.5)	0.259	1.000
Disorientation	3 (30)	4 (10)	0.103	1.000
Ataxia	0	16 (40)	**0.015**	0.375
Seizure	3 (30)	0	**0.000**	**0.000**
Nausea	7 (70)	24 (60)	0.560	1.000
Vomiting	8 (80)	22 (55)	0.149	1.000
Diarrhea	4 (40)	9 (22.5)	0.259	1.000
Abdominal pain	4 (40)	8 (20)	0.185	1.000
Rash	5 (50)	22 (55)	0.777	1.000
Pruritus	5 (50)	21 (52.5)	0.887	1.000
Bleeding	1 (10)	11 (27.5)	0.246	1.000
Conjunctivitis	5 (50)	15 (37.5)	0.470	1.000
Fatigue	6 (60)	27 (67.5)	0.650	1.000

Bold values are the *P* values which were found statistically significant. *Categorical variables are indicated as actual number and percentage, and continuous variables are indicated as median (interquartile range).

The leukocyte count and other differential white blood cell counts, excluding eosinophils, tended to be slightly higher in children than in adults, whereas the platelet counts were lower in adults, although we did not find any statistically significant differences in numbers of white blood cells, red blood cells, and platelets. Most liver function parameters were slightly elevated in children. The median albumin levels in both children and adults were in the normal range, but the median albumin level in children (4.5 g/dL; IQR: 4.2–9.15 g/dL) tended to be higher than in adults (*P* = 0.011; adjusted *P* = 0.286). Similarly, although the median globulin levels in both children and adults were within the normal range, the levels tended to be lower in children (2.25 g/dL; IQR: 0.8–2.9 g/dL) compared with adults (3 g/dL; IQR: 2.4–3.4 g/dL) (*P* = 0.033; adjusted *P* = 0.858). Regarding liver enzymes, alkaline phosphatase (ALP) levels were in the normal ranges for both children and adults. A slight elevation in aspartate aminotransferase beyond the upper normal range was observed in children and adults, with a median of 47 IU/L (IQR: 20–88 IU/L) and 38 IU/L (IQR: 29–47 IU/L), respectively. Similarly, gamma-glutamyl transpeptidase levels were slightly elevated beyond the normal range. The median gamma-glutamyl transpeptidase level was 46 IU/L (IQR: 16–167 IU/L) in children and 50 IU/L (IQR: 33–77 IU/L) in adults. Nevertheless, none of the liver enzymes, including other parameters examined in the biochemical profile, such as blood urea nitrogen, C-reactive protein, and creatinine kinase levels, differed significantly between children and adults. However, the median serum creatinine level in adults (1.08 mg/dL; IQR: 0.77–1.35 mg/dL) was elevated compared with the median level in children (0.57 mg/dL; IQR: 0.51–0.70 mg/dL) (*P* = 0.002; adjusted *P* = 0.052), as shown in Table 2.

**Table 2:** Laboratory findings of acute chikungunya in children and adults (*N* = 50)

Laboratory profile	Children (*N* = 10)		Adults (*N* = 40)		*P* value	Adjusted *P* value
	*n*	Median (IQR)	*n*	Median (IQR)		
Ct value	10	19.63 (18.01–24.64)	40	23.55 (17.93–30.68)	0.159	1.000
Leukocytes/µL	10	7,590 (4,300–9,042)	39	6,200 (4,365–7,470)	0.184	1.000
Neutrophils/µL	10	4,093 (2,631–6,837)	39	3,230 (2,582–4,210)	0.472	1.000
Lymphocytes/µL	10	1,093 (661–3,065)	39	948 (572–1,690)	0.286	1.000
Monocytes/µL	10	747 (281–966)	39	507 (391–629)	0.372	1.000
Eosinophils/µL	10	15 (7–132)	39	21 (5–84)	0.980	1.000
Basophils/µL	10	32 (6–94)	39	12 (0–36)	0.125	1.000
Hemoglobin g/dL	10	12.65 (11.15–13.85)	40	12.81 (11.30–13.85)	0.689	1.000
Hematocrit (%)	10	37.90 (34.65–43.60)	40	38.40 (33.63–41.48)	0.743	1.000
Platelets (10^3^/µL)	10	191 (165–296)	40	166 (139–196)	0.087	1.000
Total bilirubin (mg/dL)	8	0.9 (0.4–3.3)	29	0.5 (0.3–4.1)	0.372	1.000
Direct bilirubin (mg/dL)	8	0.4 (0.2–32)	29	0.2 (0.2–0.3)	0.454	1.000
Total protein (g/dL)	8	7.5 (7.0–13.4)	28	7 (6.1–7.7)	0.127	1.000
Albumin (g/dL)	8	4.5 (4.2–9.15)	27	4.1 (3.9–4.4)	**0.011**	0.286
Globulin (g/dL)	8	2.25 (0.8–2.9)	25	3 (2.4–3.4)	**0.033**	0.858
Albumin/globulin ratio	8	1.61 (1.45–2.02)	27	1.34 (1.22–1.58)	**0.054**	1.000
ALP (IU/L)	9	165 (1.5–286)	29	65 (57–88)	0.110	1.000
AST (IU/L)	9	47 (20–88)	32	38 (29–47)	0.765	1.000
ALT (IU/L)	9	35 (23–44)	32	38 (23–49)	0.682	1.000
GGT (IU/L)	7	46 (16-167)	25	50 (33–77)	0.802	1.000
BUN (mg/dL)	5	11 (9.5–14)	24	15 (10.2–18.7)	0.184	1.000
Creatinine (mg/dL)	5	0.57 (0.51–0.70)	30	1.08 (0.77–1.35)	**0.002**	**0.052**
CRP (mg/dL)	7	3.26 (0.95–4.61)	33	2.88 (1.5–5.02)	0.957	1.000
LDH	0	0	4	375 (368–388)	NA	NA
Creatinine kinase	1	1,706 (1,706–1,706)	6	381 (88–1,108)	0.317	1.000
INR	0	0	3	14.92 (1.15)	NA	NA

ALP = alkaline phosphatase; ALT = alanine transaminase; AST = aspartate aminotransferase; BUN = blood urea nitrogen; CRP = C-reactive protein; Ct = cycle threshold; GGT = gamma-glutamyl transpeptidase; INR = international normalized ratio; LDH = lactate dehydrogenase. Bold values are the *P* values which were found statistically significant.

### Analysis of arthritis in adults with and without comorbidities.

Patients with and without arthritis were segregated based on underlying comorbidities because chikungunya infection in individuals with underlying comorbidities might have been shown to cause severe disease. The median cycle threshold in group B patients with arthritis (18.83; IQR: 17.09–27.20) tended to be lower compared with group B patients without arthritis (29; IQR: 21.95–35.16) (*P* = 0.015; adjusted *P* = 0.165). Arthralgia was reported by all patients with arthritis in both groups A and B, and the difference with nonarthritic patients was statistically significant (*P* = 0.001; adjusted *P* = 0.011). Similarly, other arthritic manifestations, such as joint stiffness (83%) and swelling of the joints (100%), were significantly more frequent in patients with arthritis in both groups A and B. Symptoms such as headache (87.5%), rash (75%), and pruritus (68.8%) were significantly more common in patients with arthritis in group B only. Similarly, conjunctivitis and fatigue generally occurred only in patients with arthritis in both groups A and B. The only differences observed in laboratory findings involved liver enzymes. These included a difference in median aspartate aminotransferase level between patients with arthritis in group A (44 IU/L; IQR: 34.5–70 IU/L) and those without arthritis (30 IU/L; 25th percentile: 26 IU/L) (*P* = 0.042; adjusted *P* = 0.462). The median ALP in group B patients without arthritis (106 IU/L; IQR: 72.5–132.5 IU/L) was significantly elevated compared with patients with arthritis (57 IU/L; IQR: 38.75–74.5 IU/L) (*P* = 0.001; adjusted *P* = 0.011), as shown in Supplemental Table 2.

### Analysis of severe infection in children (*N* = 10).

Complications of severe CHIKV infection, such as encephalitis, occurred in three children. None of the children with severe infection exhibited arthralgia, whereas all children with non-severe infection had arthritis. Similarly, rash, pruritus, and conjunctivitis occurred in 71.4% of all children with non-severe infections. The median lymphocyte counts were 821/µL (IQR: 550–1,335/µL) in children with severe infection and 1,241/µL (IQR: 894-2530/µL) in children with non-severe infection (*P* = 0.016; adjusted *P* = 0.176). The median ALP level in children with severe infection was significantly lower (60 IU/L; IQR: 47–74 IU/L) than in children with non-severe infection (116 IU/L; IQR: 70–206 IU/L) (*P* = 0.004; adjusted *P* = 0.044). The median serum creatinine level in children with severe infection was significantly higher (1.21 mg/dL; IQR: 0.81–1.4 mg/dL) than in children with non-severe infection (0.8 mg/dL; IQR: 0.59–1.11 mg/dL). Similarly, the median C-reactive protein level tended to be higher in children with severe infection (3.94 mg/dL; IQR: 1.91–5.71 mg/dL) than in children with non-severe infection (2.62 mg/dL; IQR: 0.83–3.71 mg/dL) (*P* = 0.036; adjusted *P* = 0.396), as shown in Supplemental Table S3.

### Phylogenetic analysis of the recent Maldives CHIKV circulating in 2019.

In the present study, the CHIKV E1 protein–encoding region was directly sequenced from real-time RT-PCR CHIKV-positive patient serum. The newly obtained Maldives CHIKV sequences showed a high degree of similarity to East Central South African (ECSA) CHIKVs from India, Kenya, Pakistan, Bangladesh, Myanmar, and Thailand reported between 2014 and 2019. To further genetically characterize the recent Maldives CHIKVs, a ML phylogenetic tree was constructed with similar CHIKVs from National Center for Biotechnology Information Nucleotide Blast search results and other historical ECSA CHIKV sequences. The ML phylogeny revealed that the Maldives CHIKVs belong to the Indian Ocean Lineage (IOL) of the ESCA genotype. The Maldives viruses are closely related to the India, Kenya, and Djibouti viruses that circulated between 2014 and 2019, forming a monophyletic clade clustered together with another clade of India, Bangladesh, Pakistan, Myanmar, and Thailand viruses that circulated between 2016 and 2019. Both recent clades clustered into a larger monophyletic clade, separated from the IOL CHIKV of the previous outbreak in 2005, in which an adaptive mutation (E1-A226V) was identified. None of our CHIKVs harbored the E1-A226V mutation, but they did harbor the wild-type E1-226A instead. Furthermore, we found another mutation (E1-K211E) in CHIKVs isolated from 2014 to the present (Figure 3).

The ML phylogenetic tree was constructed based on the E1-encoding region (1,317 bp) of the CHIKV ECSA genotype using TIM2e+G4 and 1,000 μLtrafast bootstrap replicates. Bootstra*P* values (%) over 80 are labeled on each branch. The 34 Maldives CHIKVs sequenced in the present study are indicated in a solid box. One previously reported CHIKV sequence from a traveler who returned from the Maldives is shown by an asterisk. The IOL CHIKV clades E1:211E-226A and E1:211K-226A or 226V are indicated to the right. E1:211K-226V variants are indicated by dashed brackets.

## DISCUSSION

In 2019, a number of chikungunya cases were observed at the Indira Gandhi Memorial Hospital. All isolates were consistent with the ECSA genotype IOL sublineage but constituted a distinct clade from the previous IOL clade. The CHIKVs in this new clade completely lacked the E1-A226V mutation, but they had a consistent specific E1-K211E mutation. Chikungunya virus variants carrying the E1-226A and E1-K211E mutations were detected in outbreaks that occurred between 2014 and the present, particularly in India, Pakistan, Bangladesh, Myanmar, and Thailand.^5^^,^^35^^–^^37^

After the massive outbreak in 2006, sporadic cases of CHIKV infection were reported to the Health Protection Agency, and chikungunya cases were exported from the Maldives via travelers.^38^ The outbreak of 2019 was the second-largest CHIKV outbreak recorded in the Maldives since 2006.^25^ This outbreak could have been initiated by a viremic traveler and perhaps reached epidemic proportions due to interplay between the vector and intra-atoll travel by the local population. The total number of cases was markedly reduced across the country, which could have been related to several factors, such as existing protective immunity against the virus, an increase in the population over the past 13 years, under-reporting of cases, or better vector control. In contrast to the 2006 outbreak, in which some atolls (Faafu atoll) were minimally affected, all of the Maldive atolls were affected by the 2019 outbreak and reported cases of chikungunya. Overall, a greater number of cases in the 2019 outbreak were reported in the Raa and Gaaf atolls and the capital, Malé, than in the rest of the country. Further, the incidence differed between the northern and southern parts of Gaaf atoll, reflecting the factors mentioned above.

This study found that males were predominantly affected by the CHIKV outbreak, in contrast to previous studies reporting female sex as a risk factor for infection. We also observed that females with chikungunya exhibited more arthritogenic symptoms compared with the males in our previous work.^14^ Overall, 24.4% of children were reportedly affected during the 2019 outbreak in the Maldives, and in our cohort, we observed 20% with confirmed infection, compared with 17% of children reportedly affected in an outbreak on the Indian subcontinent.^25^^,^^39^ These data indicate that the approximate proportion of children affected during outbreaks is generally less than one-fourth of all cases. We also observed similarity between the mean age of children affected in our cohort and data reported regarding an outbreak in South America.^40^

As previously reported, the severity of CHIKV infection increases with age, and the current literature indicates that underlying comorbidities contribute to the severity of infection.^41^ In our cohort, one of the elderly patients developed sepsis and disseminated intravascular coagulation, whereas three children developed encephalitis. Severe neurologic manifestations reported during the Indian Ocean CHIKV outbreak in 2005 commonly involved children.^17^^,^^42^ Children tend to present to the hospital earlier during the course of illness than adults. This might be attributed to concerned parents bringing their child to the hospital due to the persistent high-grade fever and rash that follow the onset of viral illness because dengue is endemic in the Maldives and can have a more severe outcome.

Our findings of CHIKV symptoms were consistent with those previously reported.^6^^,^^12^ Arthralgia and myalgia occurred in 82% of cases. This mirrors the pathogenesis of CHIKV infection because the virus replicates in tissues and cells of the musculoskeletal system.^43^ Our analysis also revealed that arthritogenic symptoms occur in all age groups, and in adults, the frequency of these manifestations was significantly higher than that of children. Similarly, arthritogenic manifestations are reportedly much less common in children than in adults.^39^ This might be due to the robust nature of the immune system of children, which enables more rapid clearance of the infection.^44^ Febrile exanthema is a challenge for clinicians because a wide range of viral and bacterial infections can produce a rash. The cutaneous involvement in CHIKV infection has been well described, and some rashes that occur in other conditions, such as hyperpigmentation, scar phenomenon, and erysipelas of the pinnae (Milians ear sign), have been reported in CHIKV infection.^13^^,^^45^^,^^46^ Half of the cases in the present study developed rash, commonly a maculopapular rash, which was associated with pruritus, and one patient exhibited the Milians ear sign and scar phenomenon.

Neurologic complications such as encephalitis were observed up to 30% of children in the present study, and cerebral ataxia resulting in limited ambulatory function occurred only in adults. Although CHIKV is not a neurotropic virus, these phenomena have been reported with CHIKV infection, similar to infection with other viruses.^47^^,^^48^ We observed some discrepancies between children and adults with regard to some biochemical profile parameters upon presentation to the hospital. The median albumin level was significantly lower in adults compared with children, whereas the median globulin level was significantly higher in adults. The inflammatory process during arthritis, observed in adult cases, can cause such a decrease in albumin levels, whereas the low levels of serum globulin in children could have been associated with the increased albumin to globulin ratio.

We previously demonstrated that arthralgia and a decrease in lymphocyte count are associated with higher viremia.^49^ In the present study, we also observed a lower number of circulating lymphocytes in patients with arthritis, along with a lower cycle threshold value and other signs of systemic involvement and inflammation. The elevated serum creatinine levels in adults could have been due to worsening kidney function associated with pre-existing comorbidities. Nevertheless, increased levels of serum creatinine were observed in children and adults related to acute interstitial nephritis.^50^

We took this opportunity to compare the clinical and laboratory profiles of patients in the present and previous CHIKV infection studies with our dengue patient cohort.^34^^,^^49^ Early in the onset of illness, dengue cases exhibited an increased frequency of headaches compared with both CHIKV patient cohorts. Decreases in the number of circulating leukocytes, neutrophils, lymphocytes, and platelets were more common in dengue cases than in CHIKV infection cases in the present study. We did not observe this difference in white blood cell indices in our previous study. Nevertheless, in both CHIKV patient cohorts, hemoglobin levels were lower than those of dengue patients, which could be due to the hemoconcentration frequently seen in dengue. On the fourth and fifth days of illness, hemoglobin and hematocrit levels were lower in patients in the present study, whereas dengue patients remained symptomatic with more frequent headaches and bicytopenia than patients in both CHIKV cohorts. In resource-limited settings where rapid diagnostic tests are not readily and widely available, it would be difficult to distinguish the clinical symptoms of these infections because they are similar at presentation. However, evaluation of the above-mentioned parameters might aid clinicians in narrowing the differential diagnosis to dengue in suspected CHIKV infections.

There were some limitations in this study, one of which was the small sample size, which might limit the generalizability of our results. Further, there were no longitudinal data, which would provide information regarding the persistence of symptoms after the acute phase. More prospective studies will be required to validate our findings in greater detail and enhance understanding of the morbidity and impact of CHIKV infection on patients.

## CONCLUSION

We demonstrated that the 2019 CHIKV outbreak in the Maldives grew to epidemic proportions and affected all of the country’s atolls. The causative isolate was found to be endemic, with a distinct lineage geographically limited to the Maldives. Analyses of a subset of cases that occurred during this outbreak revealed classical features of CHIKV infection, such as arthralgia and arthritis, which were more common in affected adults than children, with encephalitis developing only in children. Arthritides in adults occurred irrespective of underlying comorbidities and was associated with the degree of viremia.

## Supplemental Materials


Supplemental materials
