# Supplementary figures and images for: Clinical Features of Acute Chikungunya Virus Infection in Children and Adults during an Outbreak in the Maldives
Source: Am J Trop Med Hyg. 2021 Aug 2;105(4):946–54. doi: 10.4269/ajtmh.21-0189 (PMC8592165; doi:10.4269/ajtmh.21-0189)

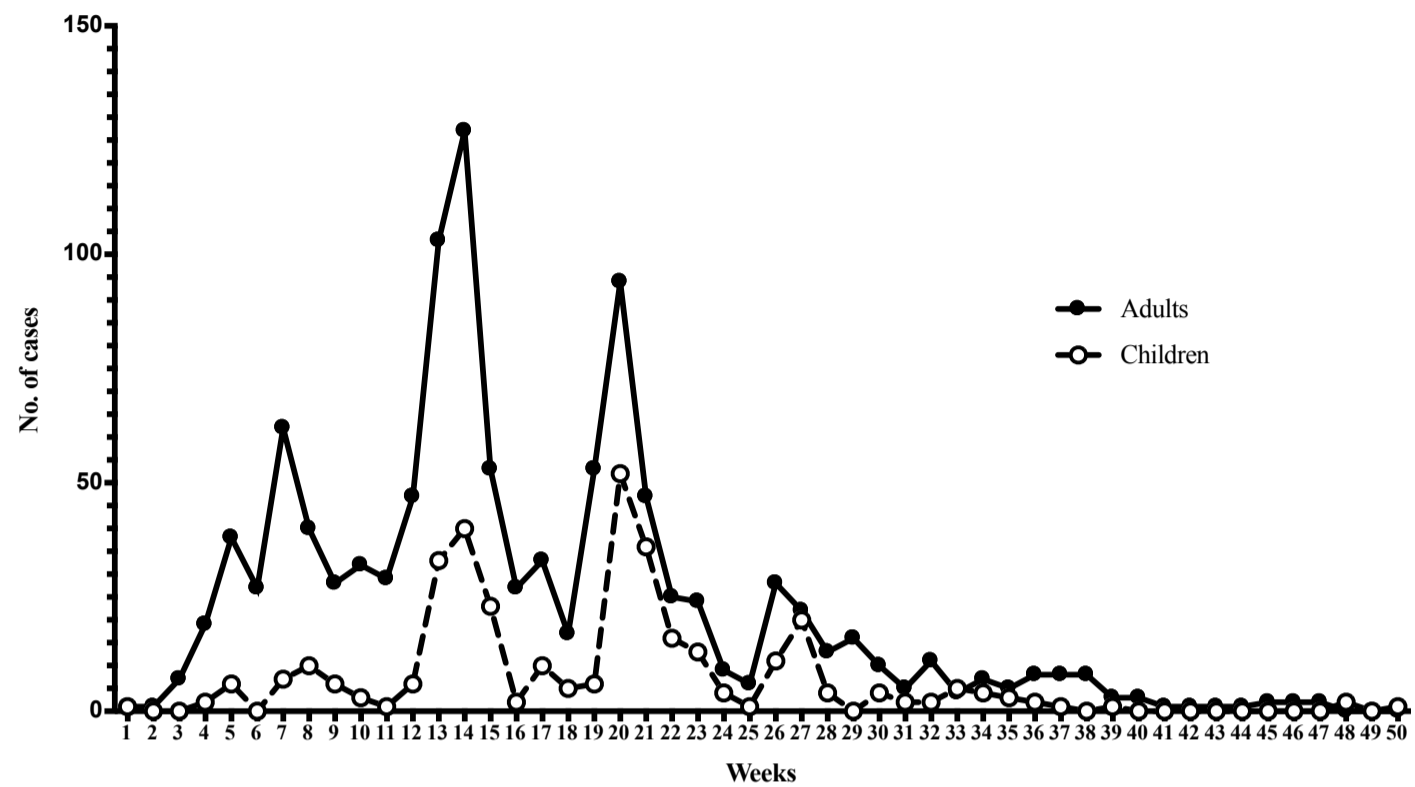

Supplement: Supplementary file 1 [file tpmd210189.SD1.pdf]

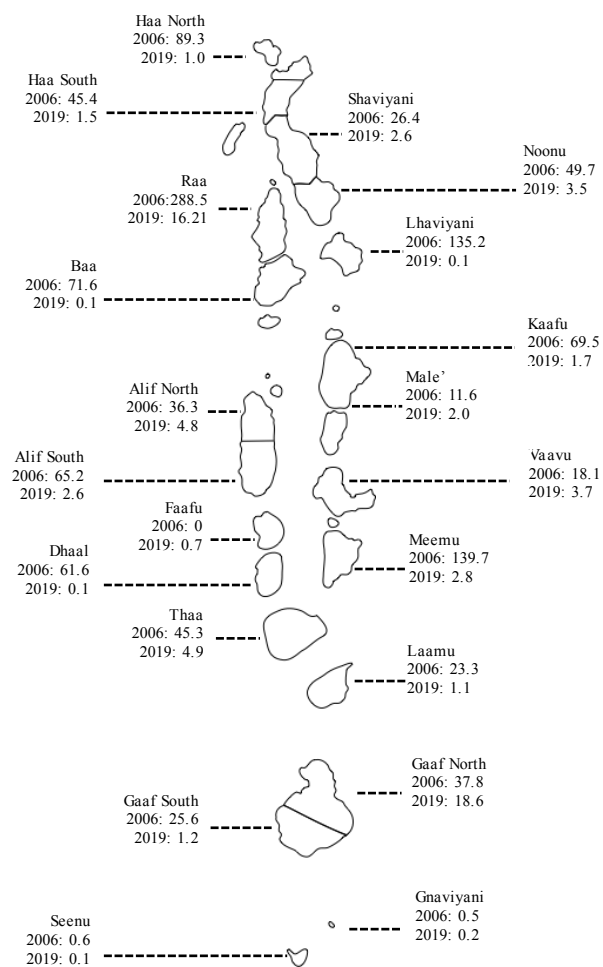

Cases per 1000 person years

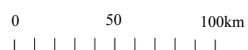

Supplement: Supplementary file 2 [file tpmd210189.SD2.pdf]

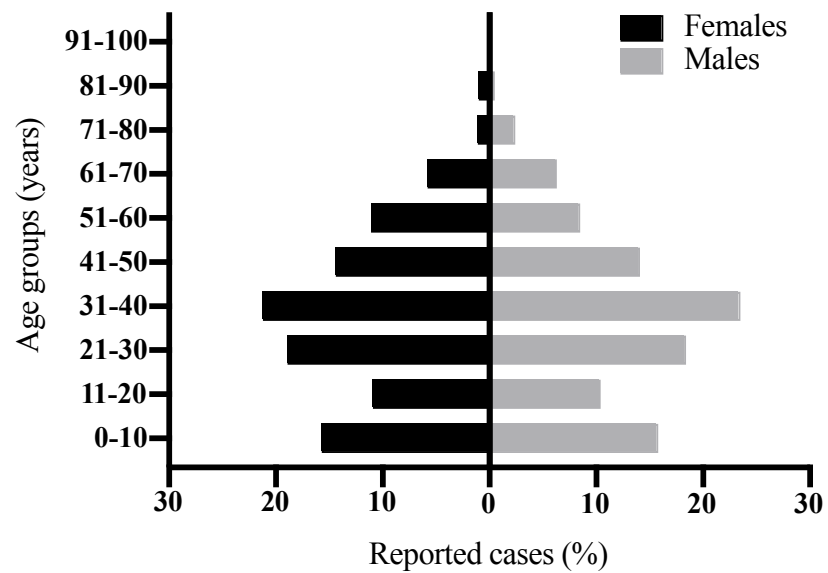

Supplement: Supplementary file 3 [file tpmd210189.SD3.pdf]
