# Supplementary material for: Clinical Features of Acute Chikungunya Virus Infection in Children and Adults during an Outbreak in the Maldives
Source: Am J Trop Med Hyg. 2021 Aug 2;105(4):946–54. doi: 10.4269/ajtmh.21-0189 (PMC8592165; doi:10.4269/ajtmh.21-0189)
Supplement: Supplementary file 4 [file tpmd210189.SD4.pdf]

Supplementary Materials

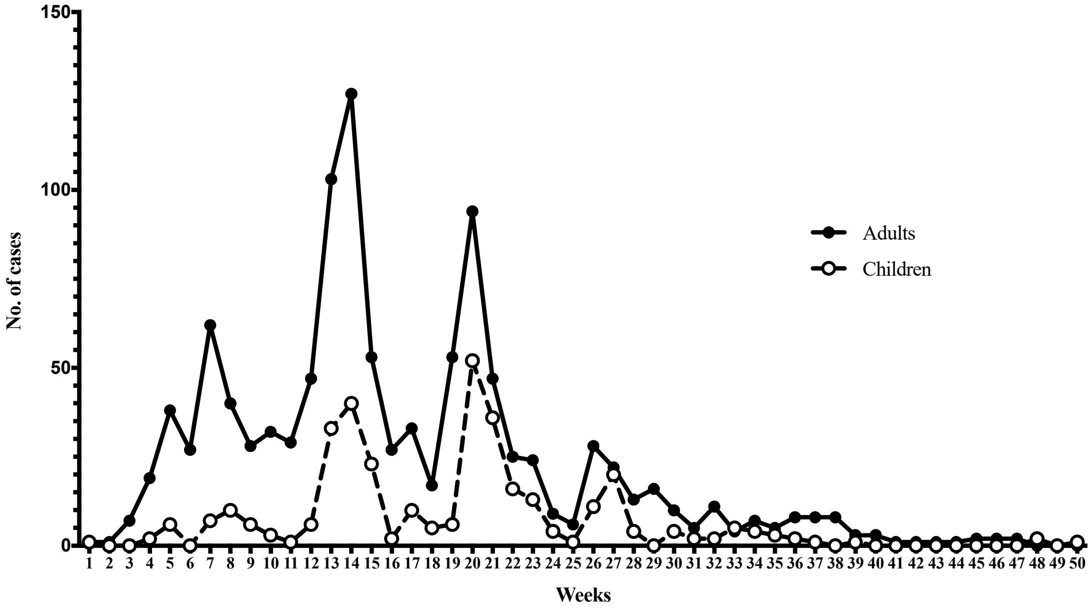

Figure S1. Cases of chikungunya reported to the Health Protection Agency, Ministry of Health, in the Maldives in 2019.

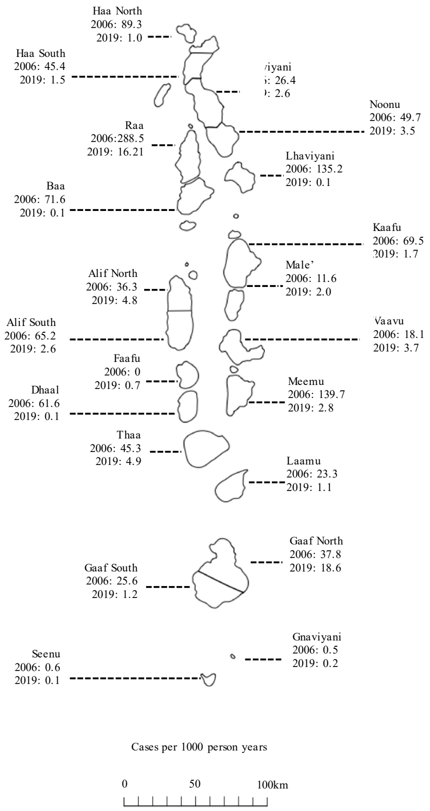

Figure S2. Map of the Maldives, depicting the incidence rates of chikungunya within the atolls during the 2006 and 2019 outbreaks. This figure was created using the incidence rates for 2006 from Yoosuf et al., 2008 (doi: 10.1016/j.trstmh.2008.09.006) and data reported to the Health Protection Agency, Ministry of Health, Maldives.

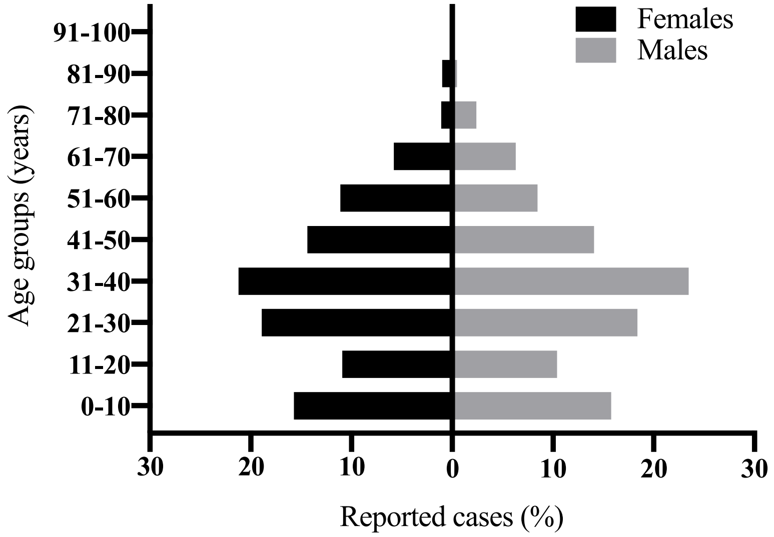

Figure S3. Gender distribution among cases of chikungunya reported to the Health Protection Agency, Ministry of Health, in the Maldives in 2019 (n = 1470).

Table S1. Performance of the immunochromatography test kit used in this study.

**Diagnostic**	**Confirmed**	**Suspected**
Antigen positive	35	4*
Antigen negative	15	13
Real-time RT-PCR positive	50	0
**Statistic**	**Value**	
Sensitivity	70.0%	
Specificity	81.25%	
Positive predictive value	92.10%	
Negative predictive value	46.42%	

*One antigen-positive case in the suspected case group was not included in the calculation of sensitivity, specificity, and positive and negative predictive values, as this case was not tested by real-time RT-PCR. Formulas: sensitivity = true positive/(true positive + false negative) × 100; specificity = true negative/(true negative + false positive) × 100; Positive predictive value = true positive/(true positive + false positive) × 100; Negative predictive value = true negative/(true negative + false negative) × 100.

Table S2. Association between arthritis and chikungunya in adults with and without comorbidities

	**Group A**				**Group B**			
	Arthritis = 12	No arthritis = 3	p value	adjusted p	Arthritis = 16	No arthritis =9	p value	adjusted p
Ct value	26.42 (17.69-30.50)	33.93 (23.08)	0.168	1.000	18.83 (17.09-27.20)	29 (21.95-35.16)	**0.015**	0.165
Arthralgia	12 (100)	2 (66.7)	**0.038**	0.418	16 (100)	4 (44.4)	**0.001**	**0.011**
Joint swelling	10 (83)	0	**0.006**	0.066	11 (68.8)	0	**0.001**	**0.011**
Joint stiffness	12 (100)	0	**0.000**	**0.000**	14 (87.5)	0	**0.000**	**0.000**
Headache	11 (91.7)	2 (66.7)	0.255	1.000	14 (87.5)	4 (44.4)	**0.021**	0.231
Rash	6 (50)	2 (66.7)	0.605	1.000	12 (75)	2 (22.2)	**0.001**	**0.011**
Pruritus	6 (50)	2 (66.7)	0.605	1.000	11 (68.8)	2 (22.2)	**0.021**	0.231
Conjunctivitis	8 (66.7)	0	**0.038**	0.418	7 (43.8)	0	**0.019**	0.209
Fatigue	10 (83.3)	0	**0.006**	0.066	14 (87.5)	3 (33.3)	**0.005**	**0.055**
AST	44 (34.5-70)	30 (26)*	**0.042**	0.462	33 (20-40.5)	42 (32-81)	0.120	1.000
ALP	60 (58-79.5)	75 (51)*	0.734	1.000	57 (38.75-74.5)	106 (72.5-132.5)	**0.011**	0.121

Group A, with underlying comorbidities (n = 15); group B, without underlying comorbidities (n = 25).

Ct: cycle threshold; AST: aspartate aminotransferase; ALP: alkaline phosphatase.

*: Due to the small number of this group, only 25th percentiles are shown for these parameters.

Table S3. Clinical findings in children with severe and non-severe chikungunya

	**Severe infection (n = 3)**	**Non-severe infection (n = 7)**	**p value**	**adjusted p**
Ct value	19.75 (19.52)*	18.63 (16.84-24.79)	0.569	1.000
Hospitalization	3 (100)	0	**0.002**	**0.022**
Arthralgia	0	7 (100)	**0.002**	**0.022**
Disorientation	3 (100)	0	**0.002**	**0.022**
Rash	0	5 (71.4)	**0.038**	0.148
Pruritus	0	5 (71.4)	**0.038**	0.418
Conjunctivitis	0	5 (71.4)	**0.038**	0.418
Lymphocytes/µL	821 (550-1335)	1241 (894-2530)	**0.016**	0.176
ALP (IU/L)	60 (47-74)	116 (70-206)	**0.004**	**0.044**
Creatinine (mg/dL)	1.21 (0.81-1.4)	0.8 (0.59-1.11)	**0.013**	0.143
CRP (mg/dL)	3.94 (1.91-5.71)	2.62 (0.83-3.71)	**0.036**	0.396

Ct: cycle threshold; ALP: alkaline phosphatase; CRP: C-reactive protein; *Due to the small number of this group, only 25th percentile is shown for this parameter.

Table S4. Comparison of clinical and laboratory findings between chikungunya and dengue.

**Group A**	**Chikungunya (n = 17)**	**Dengue (n = 9)**	**p value**	**adjusted p**
Headache	10 (58.8)	9 (100)	**0.024**	0.240
Myalgia or arthralgia	13 (76.5)	8 (88.9)	0.445	1.000
Rash	9 (52.9)	4 (44.4)	0.114	1.000
Bleeding	4 (23.5)	0	0.680	1.000
Leukocytes/µL	6520 (4960-8590)	3800 (2730-4850)	**0.006**	0.060
Neutrophils/µL	4698 (2371-6126)	2590 (1667-3445)	**0.004**	**0.040**
Lymphocytes/µL	1118 (736-1965)	560 (337-774)	**0.003**	**0.030**
Hemoglobin (g/dL)	12.8 (11-13.5)	14.1 (12.6-15.5)	**0.029**	0.290
Hematocrit (%)	38 (31-42)	40.5 (36.8-45.2)	0.153	1.000
Platelets (10^3/µL)	188 (158-227)	136 (78 -190)	**0.022**	0.220
**Group B**	**Chikungunya (n = 26)**	**Dengue (n = 22)**	**p value**	**adjusted p**
Headache	24 (88.9)	20 (90.2)	0.816	1.000
Myalgia or arthralgia	25 (92.6)	19 (86.4)	0.474	1.000
Rash	16 (59.3)	12 (54.5)	0.740	1.000
Bleeding	8 (29.6)	5 (22.7)	0.586	1.000
Leukocytes/µL	5400 (3355-8200)	3000 (2375-4200)	**0.000**	**0.000**
Neutrophils/µL	3703 (2058-6336)	1659 (1214-2436)	**0.001**	**0.010**
Lymphocytes/µL	923 (598-1747)	619 (421-997)	**0.027**	0.270
Hemoglobin (g/dL)	13.2 (11.3-15)	13.9 (12.3-15.0)	0.228	1.000
Hematocrit (%)	38 (34-42)	41.1 (37.48-43.58)	0.117	1.000
Platelets (10^3/µL)	167 (133-198)	87 (62-127)	**0.000**	**0.000**
**Group C**	**Chikungunya (n = 6)**	**Dengue (n = 67)**	**p value**	**adjusted p**
Headache	3 (50)	61 (91)	**0.003**	**0.030**
Myalgia or arthralgia	6 (100)	52 (77.6)	0.194	1.000
Rash	2 (33.3)	33 (49.3)	0.159	1.000
Bleeding	0	17 (25.4)	0.455	1.000
Leukocytes/µL	3495 (2610-5280)	3000 (2300-4200)	0.463	1.000
Neutrophils/µL	2268 (1692-2450)	1413 (1071-1975)	0.012	0.120
Lymphocytes/µL	1018 (397-1611)	841 (528-1252)	0.967	1.000
Hemoglobin (g/dL)	12.7 (10.48-13.48)	14.5 (13.5-15.6)	**0.007**	0.070
Hematocrit (%)	38 (33.25-41)	42.9 (40-46.4)	**0.018**	0.180
Platelets (10^3/µL)	155 (128-182)	58 (30-86)	**0.000**	**0.000**

- Group A: patients who presented on days 1 and 2 of illness; Group B: patients who presented on day 3 of illness; Group C: patients who presented on days 4 and 5 of illness. Data regarding the dengue patient cohort were obtained from our previous publication, Imad et al., 2020 (doi: 10.4269/ajtmh.19-0487).

Note added to Table S4.

Both chikungunya and dengue exhibit similar clinical manifestation and the basic laboratory profile cannot distinguish one from the other. We therefore took this opportunity to compare the common clinical findings and basic laboratory profile at the time of presentation to look for differences between these diseases. The results of a comparison of clinical and laboratory findings between CHIKV and dengue virus infections with respect to day of illness are tabulated in Table S4, since clinical and laboratory findings in these diseases greatly change during the course of infection. In group A (patients who presented on days 1 and 2 of illness), headache was significantly more frequently reported in dengue ($p = 0.024$, adjusted $P = 0.240$) than CHIKV. The median leukocyte count, neutrophils, lymphocytes, hemoglobin levels, and platelets in group A patients were significantly lower in dengue than CHIKV. In group B (patients who presented on day 3 of illness), there were no significant differences observed in the clinical manifestations between dengue and CHIKV. However, the median leukocytes, neutrophils, and platelets were significantly lower in dengue than CHIKV. In group C (patients who presented on days 4 and 5 of illness), headache was significantly more frequent, hemoglobin and hematocrit significantly increased, and thrombocytopenia significantly more common in dengue than CHIKV.
