# Supplementary material for: Clinical Features of Acute Chikungunya Virus Infection in Children and Adults during an Outbreak in the Maldives
Source: Am J Trop Med Hyg. 2021 Aug 2;105(4):946–54. doi: 10.4269/ajtmh.21-0189 (PMC8592165; doi:10.4269/ajtmh.21-0189)
Supplement: Supplementary file 5 [file tpmd210189.SD5.pdf]

Table S1. Performance of the immunochromatography test kit used in this study.

Diagnostic	Confirmed	Suspected
Antigen positive	35	4*
Antigen negative	15	13
Real-time RT-PCR positive	50	0
**Statistic**	**Value**	
Sensitivity	70.0%	
Specificity	81.25%	
Positive predictive value	92.10%	
Negative predictive value	46.42%	

*One antigen-positive case in the suspected case group was not included in the calculation of sensitivity, specificity, and positive and negative predictive values, as this case was not tested by real-time RT-PCR. Formulas: sensitivity = true positive/(true positive + false negative) × 100; specificity = true negative/(true negative + false positive) × 100; Positive predictive value = true positive/(true positive + false positive) × 100; Negative predictive value = true negative/(true negative + false negative) × 100.
