# Supplementary material for: Clinical Features of Acute Chikungunya Virus Infection in Children and Adults during an Outbreak in the Maldives
Source: Am J Trop Med Hyg. 2021 Aug 2;105(4):946–54. doi: 10.4269/ajtmh.21-0189 (PMC8592165; doi:10.4269/ajtmh.21-0189)
Supplement: Supplementary file 6 [file tpmd210189.SD6.pdf]

Table S2. Association between arthritis and CHIKV infection in adults with and without comorbidities

	Group A				Group B			
	Arthritis = 12	No arthritis = 3	p value	adjusted p	Arthritis = 16	No arthritis =9	p value	adjusted p
Ct value	26.42 (17.69-30.50)	33.93 (23.08)	0.168	1.000	18.83 (17.09-27.20)	29 (21.95-35.16)	**0.015**	0.165
Arthralgia	12 (100)	2 (66.7)	**0.038**	0.418	16 (100)	4 (44.4)	**0.001**	**0.011**
Joint swelling	10 (83)	0	**0.006**	0.066	11 (68.8)	0	**0.001**	**0.011**
Joint stiffness	12 (100)	0	**0.000**	**0.000**	14 (87.5)	0	**0.000**	**0.000**
Headache	11 (91.7)	2 (66.7)	0.255	1.000	14 (87.5)	4 (44.4)	**0.021**	0.231
Rash	6 (50)	2 (66.7)	0.605	1.000	12 (75)	2 (22.2)	**0.001**	**0.011**
Pruritus	6 (50)	2 (66.7)	0.605	1.000	11 (68.8)	2 (22.2)	**0.021**	0.231
Conjuncti vitis	8 (66.7)	0	**0.038**	0.418	7 (43.8)	0	**0.019**	0.209
Fatigue	10 (83.3)	0	**0.006**	0.066	14 (87.5)	3 (33.3)	**0.005**	**0.055**
AST	44 (34.5-70)	30 (26)*	**0.042**	0.462	33 (20-40.5)	42 (32-81)	0.120	1.000
ALP	60 (58-79.5)	75 (51)*	0.734	1.000	57 (38.75-74.5)	106 (72.5-132.5)	**0.011**	0.121

Group A, with underlying comorbidities (n = 15); group B, without underlying comorbidities (n = 25).
Ct: cycle threshold; AST: aspartate aminotransferase; ALP: alkaline phosphatase.
*: Due to the small number of this group, only 25th percentiles are shown for these parameters.
