# Supplementary material for: Clinical Features of Acute Chikungunya Virus Infection in Children and Adults during an Outbreak in the Maldives
Source: Am J Trop Med Hyg. 2021 Aug 2;105(4):946–54. doi: 10.4269/ajtmh.21-0189 (PMC8592165; doi:10.4269/ajtmh.21-0189)
Supplement: Supplementary file 7 [file tpmd210189.SD7.pdf]

Table S3. Clinical findings in children with severe and non-severe chikungunya.

	**Severe infection (n = 3)**	**Non-severe infection (n = 7)**	**p value**	**adjusted p**
Ct value	19.75 (19.52)*	18.63 (16.84-24.79)	0.569	1.000
Hospitalization	3 (100)	0	**0.002**	**0.022**
Arthralgia	0	7 (100)	**0.002**	**0.022**
Disorientation	3 (100)	0	**0.002**	**0.022**
Rash	0	5 (71.4)	**0.038**	0.148
Pruritus	0	5 (71.4)	**0.038**	0.418
Conjunctivitis	0	5 (71.4)	**0.038**	0.418
Lymphocytes/μL	821 (550-1335)	1241 (894-2530)	**0.016**	0.176
ALP (IU/L)	60 (47-74)	116 (70-206)	**0.004**	**0.044**
Creatinine (mg/dL)	1.21 (0.81-1.4)	0.8 (0.59-1.11)	**0.013**	0.143
CRP (mg/dL)	3.94 (1.91-5.71)	2.62 (0.83-3.71)	**0.036**	0.396

Ct: cycle threshold; ALP: alkaline phosphatase; CRP: C-reactive protein; *Due to the small number of this group, only 25th percentile is shown for this parameter.
