# Supplementary material for: Clinical Features of Acute Chikungunya Virus Infection in Children and Adults during an Outbreak in the Maldives
Source: Am J Trop Med Hyg. 2021 Aug 2;105(4):946–54. doi: 10.4269/ajtmh.21-0189 (PMC8592165; doi:10.4269/ajtmh.21-0189)
Supplement: Supplementary file 8 [file tpmd210189.SD8.pdf]

Table S4. Comparison of clinical and laboratory findings between chikungunya and dengue.

**Group A**	**Chikungunya (n = 17)**	**Dengue (n = 9)**	**p value**	**adjusted p**
Headache	10 (58.8)	9 (100)	**0.024**	0.240
Myalgia or arthralgia	13 (76.5)	8 (88.9)	0.445	1.000
Rash	9 (52.9)	4 (44.4)	0.114	1.000
Bleeding	4 (23.5)	0	0.680	1.000
Leukocytes/µL	6520 (4960-8590)	3800 (2730-4850)	**0.006**	0.060
Neutrophils/µL	4698 (2371-6126)	2590 (1667-3445)	**0.004**	**0.040**
Lymphocytes/µL	1118 (736-1965)	560 (337-774)	**0.003**	**0.030**
Hemoglobin (g/dL)	12.8 (11-13.5)	14.1 (12.6-15.5)	**0.029**	0.290
Hematocrit (%)	38 (31-42)	40.5 (36.8-45.2)	0.153	1.000
Platelets (10^3/µL)	188 (158-227)	136 (78 -190)	**0.022**	0.220
**Group B**	**Chikungunya (n = 26)**	**Dengue (n = 22)**	**p value**	**adjusted p**
Headache	24 (88.9)	20 (90.2)	0.816	1.000
Myalgia or arthralgia	25 (92.6)	19 (86.4)	0.474	1.000
Rash	16 (59.3)	12 (54.5)	0.740	1.000
Bleeding	8 (29.6)	5 (22.7)	0.586	1.000
Leukocytes/µL	5400 (3355-8200)	3000 (2375-4200)	**0.000**	**0.000**
Neutrophils/µL	3703 (2058-6336)	1659 (1214-2436)	**0.001**	**0.010**
Lymphocytes/µL	923 (598-1747)	619 (421-997)	**0.027**	0.270
Hemoglobin (g/dL)	13.2 (11.3-15)	13.9 (12.3-15.0)	0.228	1.000
Hematocrit (%)	38 (34-42)	41.1 (37.48-43.58)	0.117	1.000
Platelets (10^3/µL)	167 (133-198)	87 (62-127)	**0.000**	**0.000**
**Group C**	**Chikungunya (n = 6)**	**Dengue (n = 67)**	**p value**	**adjusted p**
Headache	3 (50)	61 (91)	**0.003**	**0.030**
Myalgia or arthralgia	6 (100)	52 (77.6)	0.194	1.000
Rash	2 (33.3)	33 (49.3)	0.159	1.000
Bleeding	0	17 (25.4)	0.455	1.000
Leukocytes/µL	3495 (2610-5280)	3000 (2300-4200)	0.463	1.000
Neutrophils/µL	2268 (1692-2450)	1413 (1071-1975)	0.012	0.120
Lymphocytes/µL	1018 (397-1611)	841 (528-1252)	0.967	1.000
Hemoglobin (g/dL)	12.7 (10.48-13.48)	14.5 (13.5-15.6)	**0.007**	0.070
Hematocrit (%)	38 (33.25-41)	42.9 (40-46.4)	**0.018**	0.180
Platelets (10^3/µL)	155 (128-182)	58 (30-86)	**0.000**	**0.000**

Group A: patients who presented on days 1 and 2 of illness; Group B: patients who presented on day 3 of illness; Group C: patients who presented on days 4 and 5 of illness. Data regarding the dengue patient cohort were obtained from our previous publication, Imad et al., 2020 (doi: 10.4269/ajtmh.19-0487
